# FAIR by design (TRACE): A Trusted Research Access & Collaboration Environment

**DOI:** 10.1186/s12911-026-03840-3

**Published:** 2026-09-15

**Authors:** Marcel Müller, Benjamin P. Geisler, Samar Shamas, Nikolaus v. Bomhard, Eva Hoster, Markus Pfirrmann, Ulrich Mansmann

**Affiliations:** 1 Institute for Medical Information Processing, Biometry, and Epidemiology (IBE), LMU Medizin, Ludwig-Maximilians-Universität München, Marchioninistr. 15, 81377 München, Germany; 2 Pettenkofer School of Public Health, LMU Medizin, Ludwig-Maximilians-Universität München, München, Germany; 3 Department of Health Management and Health Economics, University of Oslo, Oslo, Norway; 4 Medical Informatics Initiative, DIFUTURE, München, Germany

**Keywords:** Data sharing, TRACE, Trusted research environment, FAIR, Data privacy, Reproducibility of results, Open science, Research integrity, Information storage and retrieval

## Abstract

**Background:**

A tension exists between the open science imperative for data transparency through FAIR (Findable, Accessible, Interoperable, Reusable) data sharing and the stringent requirements of current data protection regulations for health data. Researchers lack efficient yet affordable tools to share sensitive data responsibly. Existing solutions are often either difficult to use, expensive, or lack features like variable-level access control and auditable and reproducible workflows. Our objective was to develop a solution that lowers the threshold for researchers to share sensitive data responsibly and that can be operated by existing institutional staff.

**Results:**

TRACE (Trusted Research Access & Collaboration Environment) was designed to support several data-sharing elements highlighted in the 2025 SPIRIT (Standard Protocol Items: Recommendations for Interventional Trials) and CONSORT (Consolidated Standards of Reporting Trials) statements by providing controlled and auditable workflows and supporting reproducibility at the level of data and analysis code. Its features for data governance, sharing, reproducibility and security allow institutions to manage study data access and to establish consistent, transparent standard operating procedures through granular variable selection, integrated study visualisation, and provisioning into a sealed analysis environment. TRACE provides an automated workflow for selected data-access steps that had previously relied on manual curation and ad-hoc sharing. A core feature versions datasets together with the corresponding analysis scripts, supporting reproducibility at the level of data and code; the computational environment is not captured. TRACE has supported data governance across seven clinical studies and processed 56 data access applications. Across three studies, an uncontrolled before-and-after comparison found 42.4 manual interface exports per 30 days before TRACE and 4.7 after implementation; causality cannot be inferred.

**Conclusions:**

Platforms like TRACE may help shift resource-intensive, expert-dependent data governance towards automated processes. Operated by existing institutional staff, TRACE may make controlled data sharing feasible for institutions with limited resources. By automating previously manual steps, TRACE is intended to reduce the administrative burden of governed data sharing; whether it shortens the interval from data request to analysis has not been measured. TRACE is used as a training environment for preparing and using data in sharing processes.

**Supplementary Information:**

The online version contains supplementary material available at https://doi.org/10.1186/s12911-026-03840-3.

## Background

At present, clinical researchers face a fundamental dilemma between embracing open science practices and navigating strict data privacy laws such as the European Union’s General Data Protection Regulation (GDPR) [1]. The research community’s push for transparent and Findable, Accessible, Interoperable and Reusable (FAIR) data sharing [2, 3] conflicts directly with legal and ethical obligations to protect sensitive personal information [4]. This tension leaves principal investigators asking: how can we share data responsibly without being overwhelmed by compliance overhead?

Implementing these principles in practice presents many challenges. Even pseudonymised or anonymised datasets carry re-identification risk, forcing researchers into uncomfortable trade-offs between data utility and privacy [5]. Researchers face numerous barriers to sharing data and code publicly, including uncertainty about how to prepare and archive them, concerns about subsequent reuse, and limited incentives; these barriers may contribute to many researchers not making their data and analysis code publicly available [6]. In public health, data sharing is further constrained by technical, motivational, economic, political, legal, and ethical barriers [7]. For datasets described as “available upon request,” access depends on individual request-and-response interactions that frequently do not result in data being shared and may require substantial effort from requesters [8]. Secure health-data sharing also requires appropriate security architectures, including robust authentication and access-control mechanisms, to address the complex security requirements of healthcare applications [9]. Together, these challenges motivate more structured, auditable, and secure data-sharing workflows.

Reproducible research requires access to the data, analysis code and relevant study materials used in the original analysis so that conclusions can be independently re-examined and verified [10]. This challenge has become more pressing with the new SPIRIT 2025 and CONSORT 2025 statements, which now include a dedicated open science section requiring clear protocols for sharing de-identified participant data, statistical analysis code, and metadata [11, 12]. On the other hand, meeting these emerging expectations may require legal, administrative, and technical capacities that are not yet consistently available across institutions [13, 14].

Clinical study data sharing remains administratively, legally, and technically demanding [13, 14]. In the German clinical-research setting, data-sharing arrangements may involve protracted processes to establish legal compliance, while uncertainty regarding GDPR obligations and legal liability can leave individual researchers bearing substantial responsibility [13]. Institutional governance, transparent decision-making processes, and durable, quality-controlled storage and access infrastructures are therefore needed to support clinical data sharing [13]. FAIRification of biomedical data likewise requires prospective planning, sufficient resources, appropriate technical and legal support, and careful attention to confidentiality and security [14]. Repository technology alone is insufficient: governed data sharing also depends on administrative oversight, enforceable data-use and licensing arrangements, ongoing support for researchers, and sustained data maintenance [14]. Specialized clinical data-sharing platforms such as Vivli and YODA provide governed access to clinical-trial data through centralized request and review processes (Table 3). In this selected comparison, we did not identify a platform combining local institutional hosting, researcher-driven variable selection, versioned reproducible datasets, and an integrated controlled analysis environment in a form accessible to institutions with limited technical capacity and budgets.

Based on our institutional experience and the design analysis underlying TRACE, we identified three practical constraints affecting adoption of responsible data sharing: (1) complexity: existing solutions may require specialised IT expertise and infrastructure that exceed the personnel, technical and financial capacities of small- to mid-sized research institutions; (2) external dependencies: centralized platforms require data transfer outside institutional control, conflicting with GDPR requirements for data minimization and local sovereignty; and (3) workflow mismatch: generic repository tools do not align with the granular, variable-level access control and audit requirements of clinical data governance. An affordable and secure researcher-centred solution is needed that provides granular variable-level access control, automates selected governance and provisioning steps, integrates reproducible workflows, and can be deployed by existing institutional staff, with an architecture intended to support future scaling [15].

To address this implementation gap, we developed TRACE (Trusted Research Access & Collaboration Environment), an open-source platform designed to operationalise responsible data sharing within institutional workflows. TRACE implements this approach in a single-site institutional deployment; its scalability has not yet been evaluated. Our objectives were to enable installation and maintenance by existing institutional IT and data management staff without specialized infrastructure, to provide self-service metadata exploration and variable-level data selection through researcher-facing web interfaces, and to embed auditable access control, versioned dataset provenance and restricted analysis environments that support alignment with FAIR principles and institutional GDPR-related governance requirements. We describe the implementation of TRACE at a large European academic medical center.

## Implementation

TRACE integrates open-source components to support secure, reproducible, and FAIR-aligned sharing of sensitive research data. TRACE is a custom trusted research environment implemented in Django 5.2.11 on Debian GNU/Linux; it indexes study metadata, manages access requests, and securely delivers approved datasets. Centralized authentication and authorization are provided via Lightweight Directory Access Protocol. Secure analysis takes place in the Ginko Virtual Monitor—adapted from the open-source DARTS platform [16]—which seals the infrastructure. For collaboration and controlled file exchange, TRACE relies on Nextcloud. Analytical workspaces, including RStudio and Jupyter Notebook (Python, R and more), are hosted internally and exposed through Ginko to keep data inside the environment.

### Accessing TRACE

Researchers start by visiting a publicly available study catalogue page where they can browse the available studies hosted by the institute. Data owners control study visibility: published studies appear in the public catalogue, while unpublished studies remain accessible only to the study team. Studies include an interactive study visualisation that maps out study events, instruments, and variables, providing a detailed overview of the data structure in real time to include REDCap (Research Electronic Data Capture) studies. REDCap is a web-based, metadata-driven platform for designing and managing research databases and electronic data-capture workflows [17].

#### Study information and metadata

For each study, comprehensive information is provided, including a description, implementation details, study purpose, funding sources, and links to relevant registries. Researchers can also download further metadata, such as data dictionaries, protocols, statistical analysis plans and publications.

#### Data access application and variable selection

Researchers interested in accessing data can apply through the platform, selecting individual variables from the entire study relevant to their research. Applicants must also provide a clear purpose and aim for their proposed use of the data.

#### Application review and notifications

Once submitted, the researcher receives a notification email confirming receipt of the application. The data owner or person in charge reviews the request and may reject it if the purpose is insufficiently described or if the selected variables are unsuitable. Upon approval or rejection, the applicant is notified via email.

#### Using the trusted research environment

Following approval, the applicable access route depends on the researcher’s institutional permissions. Researchers without institutional VPN access receive access to the approved extract through Ginko. Authorised internal staff and study-team members may instead retain access to the source system through the institutional intranet or VPN where the data owner approves.

#### Available tools and resources

Within the TRE, users have access to essential tools such as RStudio, Jupyter Notebook, Nextcloud, and a file browser. Data files are available through the file server, enabling direct use in analysis workflows like Jupyter Notebook and RStudio.

#### Analysis, results, and publication

After completing their analysis, researchers can extract and download results in a controlled way and can publish their findings. Their scripts and data can also be shared with other researchers via TRACE—but this requires another application which has to be approved. At present, outputs are reviewed manually by the data owner before release; an integrated disclosure-review and monitored-export workflow has not yet been implemented.

#### Ensuring reproducibility and reuse

A completed data access application can be converted into a publicly available sub-project of a study. Once published, this sub-project is listed so that other researchers can apply for access to the identical dataset, supporting transparent re-analysis. To make this possible, the original comma separated value (CSV) files are both delivered to the initial applicant’s home directory on the internal file server and archived separately for future reuse.

### Data sharing workflow: any researchers, stakeholders, and other interested parties

The data owner determines whether a study is publicly available or restricted. If the data owner chooses to make a study publicly accessible, an administrator can enable this by clicking a “publish” button, making the entire study available to the world without requiring login. In this case, sub-projects can be added openly under the study. However, anyone wishing to access TRACE itself or apply for data must still create an account.

Approved data seekers must apply for an institute account and coordinate with an administrator for approval. Once granted, the account is integrated with a lightweight directory access protocol (LDAP) authentication and includes a dedicated user home directory.

Once a researcher has been granted access and their data delivered securely, the underlying platform architecture allows TRACE to handle diverse data sources and study formats. This flexibility is achieved through TRACE’s extensible connector architecture, which standardizes and integrates metadata from multiple origins to provide consistent and comprehensive study visualisation.

### Data sharing workflow: data provider perspective

Effective data engineering and management must begin at the inception of a study and be grounded in a comprehensive understanding of the research itself [18]. It starts by reviewing the study protocol, questionnaires, or study standard operating procedures (SOPs); identifying core variables with clear definitions and how they relate to one another and to the study outcomes; understanding temporal aspects such as longitudinal follow-ups or event-based data; and mapping relationships between datasets, whether at the participant, sample, or time point level. This process makes it possible that data structures reflect the real-world logic of the study rather than being shaped by quick technical fixes. By contrast, fitting structure and metadata in retrospect often results in fragmented and inconsistent data capture, undocumented variables, incompatible coding schemes used at different times or by different people, missing contextual details such as units, measurement methods, or population definitions, and difficult, error-prone harmonization. A proactive, design-driven approach can support completeness, accuracy, and long-term usability by defining coding schemes and metadata frameworks before data collection and allowing them to evolve with the study.

FAIR principles enable effective data management [3]. Applying these principles in real-world research settings is rarely straightforward. Working with TRACE integrates FAIR principles from the earliest stages of each project and maintains them throughout the entire research lifecycle—planning, data generation, analysis, and sharing. At present, TRACE supports findability through carefully structured and published metadata and stable TRACE URIs; systematic institutional DOI assignment is planned. It supports accessibility through secure and controlled workflows, interoperability through standardised formats and, where provided by the data owner, coding schemes and community vocabularies, and reusability through comprehensive documentation and variable-level metadata within the study catalogue.

FAIR compliance exists along a continuum, influenced by the stage of the project, the type of data, and governance policies defined by data owners [19]. As projects progress, their level of FAIRness can evolve and improve. Tai et al. present FAIRification across scientific, technical, and legal or ethical dimensions, with key outputs spanning planning, data preparation, sharing, and subsequent management [14]. Their framework emphasizes starting FAIRification during study planning and continuing it throughout the data-sharing lifecycle [14]. Our single-site experience suggests that a gradual, lifecycle-spanning approach is feasible; its long-term organisational and technical sustainability has not yet been evaluated.

The core of the TRACE platform uses a modular “connector” approach (based on the strategy design pattern [20]). Connectors act as adapters, enabling seamless communication between TRACE and diverse data sources, including structured databases and simpler file-based formats. Each connector converts the data source information into a standard metadata format (JavaScript object notation - JSON) by design or per configuration (see Additional File 1) and allows to visualize a study structure on a web page. TRACE does not need to know in advance which type of data source it will encounter; instead, it “picks the right strategy” for handling each source through the appropriate connector.

For a file-based data source the configuration starts with specifying the location of the data file on the server: Defining a folder where these source files can be safely stored. But, we recommend moving the study data into a proper database system, like MariaDB or PostgreSQL (SQL, structured query language). This allows better performance and manageability. Our default is REDCap with MariaDB. The configuration (JSON) defines the overall structure of the study—for example, which events occurred (like “Start of Year” or “End of Year”), what instruments were used (such as anthropometry or vision tests), and which variables were collected. This setup makes it possible to represent complex, time-based (longitudinal) studies in a clear and consistent way. However, the connector for REDCap studies already includes metadata extraction for visualisation and does not need to be added in the admin panel, as is the case with file-based data sources.

**Fig. 1:**
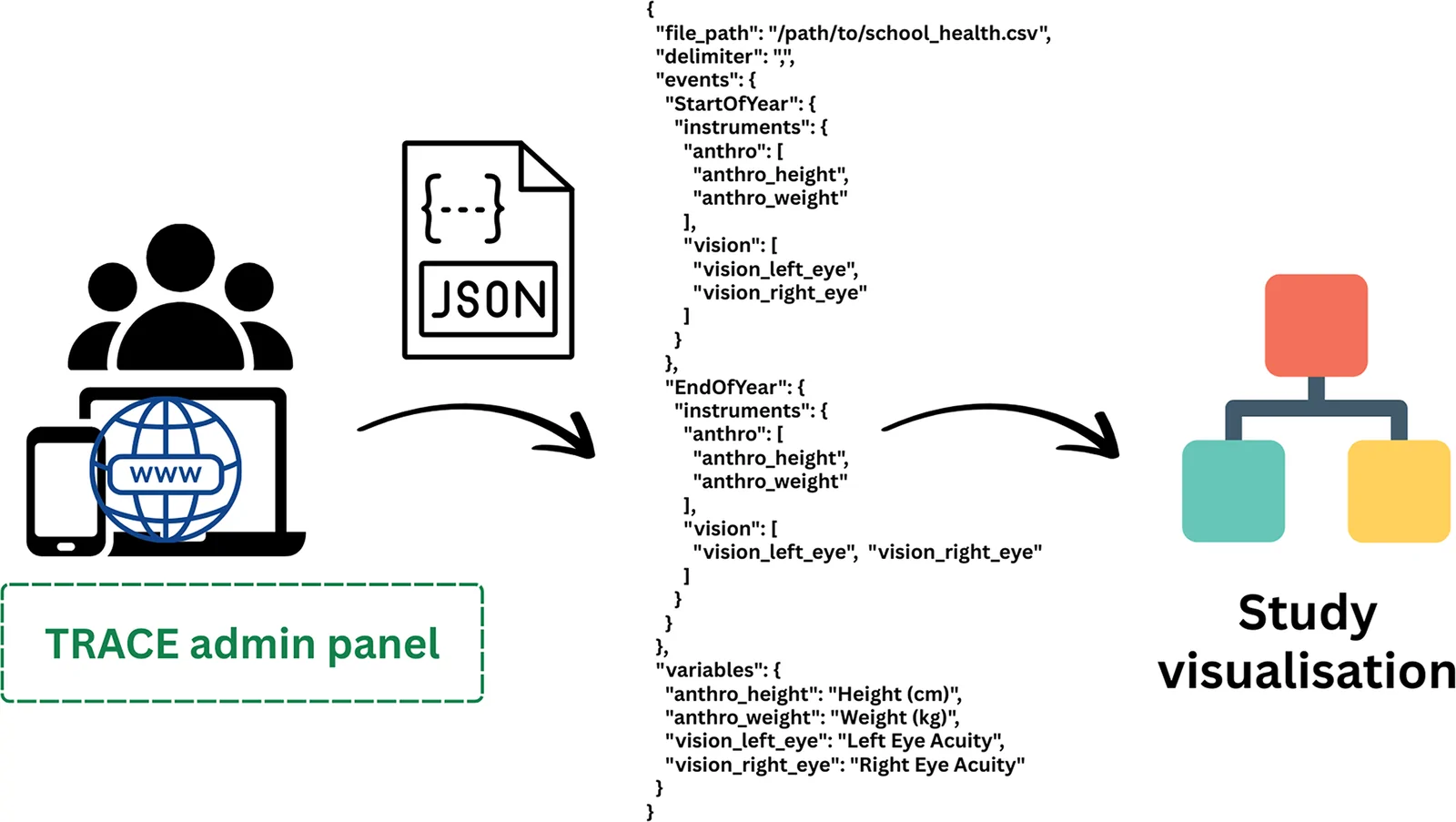
Metadata integration pathways in TRACE. Study visualisation metadata can be provided via manual JSON configuration (for file-based sources) or extracted automatically from the data source API (e.g., REDCap)

The configuration structure reflects how study metadata can be abstracted into a common format regardless of the original data source. Whether files are stored as flat CSVs or eventually migrated into a database system, the same structural pattern—defining events, instruments, and variables—can be applied as seen in Fig. 1.

TRACE uses a strategy-pattern-based implementation to support this flexibility in a maintainable and extensible way.

### Strategy pattern implementation

The connector architecture applies the **Strategy Pattern** to enforce a clean separation of concerns. It defines an abstract base class, *StudyBackend*, which standardizes the interface for all study data sources, like a REDCap project or a CSV file containing study data.

**Code snippet 1** Abstract Strategy (StudyBackend)

**Figure:**
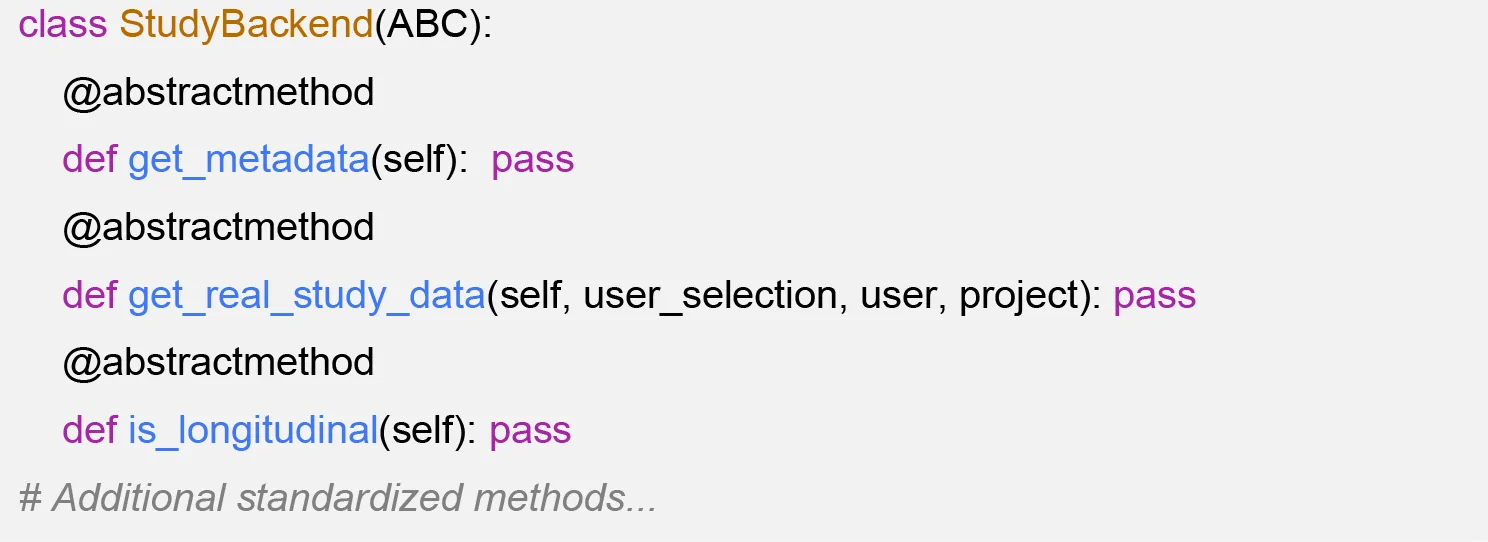

Implementations include *RedcapBackend*, which integrates with the REDCap Application Programming Interface (API) and provides full support for longitudinal studies, and *CsvBackend*, which handles flat-file data sources such as CSVs with manually defined configurations. The system is designed to be extensible, supporting the future integration of additional data sources such as fast healthcare interoperability resources (FHIR), extensible markup language (XML), and direct database connections.

**Code snippet 2** Context (Factory Pattern)

**Figure:**
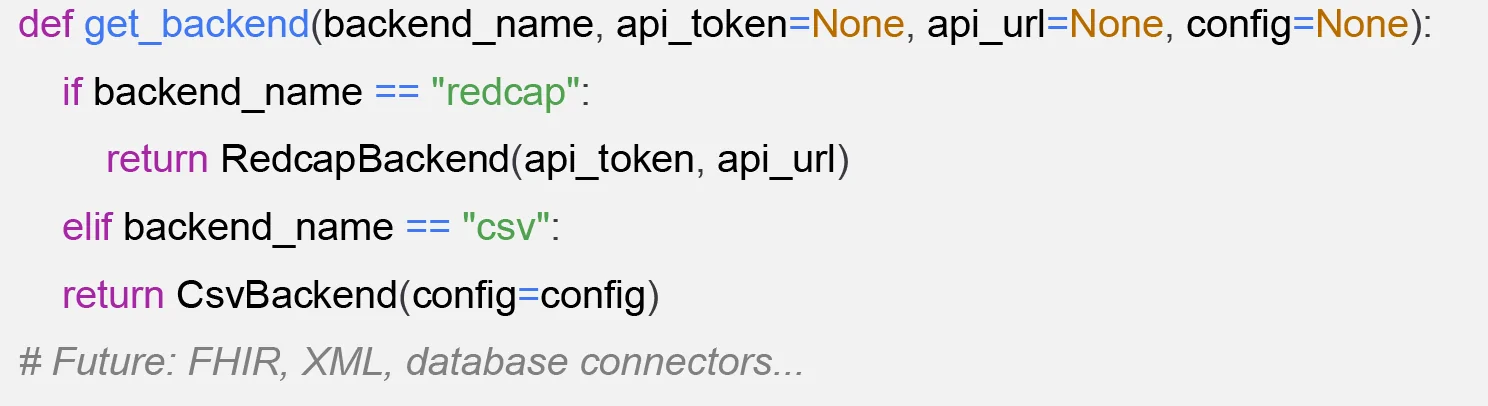

#### Data extraction algorithm

The REDCap connector implements a multi-phase extraction algorithm designed to support both longitudinal and cross-sectional study designs. The process consists of three main phases: metadata discovery, data structure harmonization, and secure data extraction.

During the metadata discovery phase, the algorithm first identifies the project type—studies can be longitudinal or not— using the is_longitudinal() method. It then extracts the complete metadata schema using the get_metadata() method, before mapping events to instruments in the case of longitudinal studies. This phase also involves identifying the relationships between variables and their associated instruments.

The data structure harmonisation phase is responsible for unifying the layout of REDCap’s hierarchical data. This is achieved using the method shown in code snippet 3.

**Code snippet 3** Data harmonization

**Figure:**
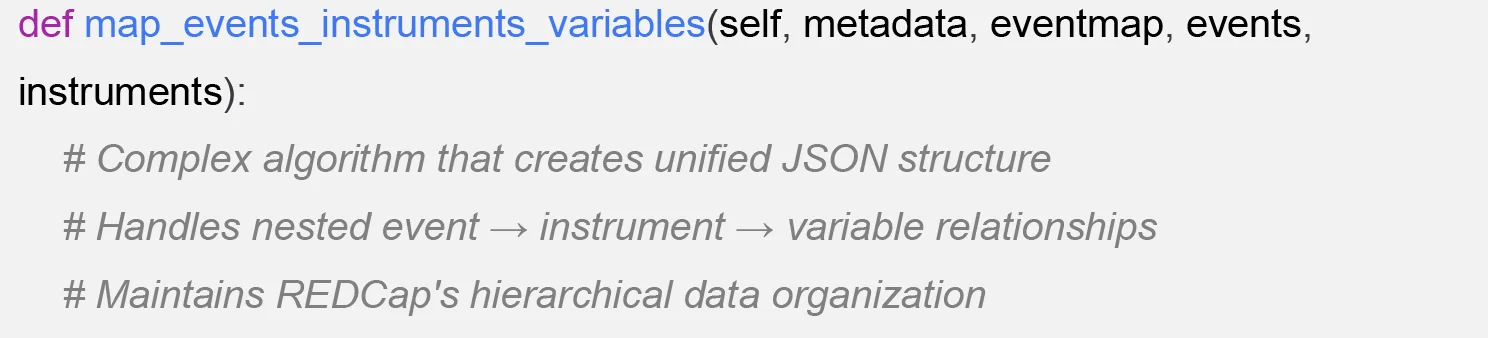

This function produces a consistent JSON representation that preserves the nested structure of events, instruments, and variables. In the secure data extraction phase, the system dynamically selects fields based on user permissions and applies event filtering for longitudinal datasets. The final output is generated in a standardized CSV format that includes metadata preservation. Additionally, each extraction is logged with an audit trail including timestamps and user identifiers, to support traceability and compliance with applicable data-governance standards.

#### Algorithmic complexity considerations

The REDCap connector addresses the structural complexity of longitudinal and cross-sectional study designs. In longitudinal projects, REDCap organizes data using a hierarchical model where events represent temporal measurement points, instruments are linked to specific events, and variables are grouped under instruments. The connector maps these relationships to support precise variable selection and data extraction, even when researchers request subsets across multiple events.

For data extraction, the connector uses the REDCap API to retrieve records in CSV format. This response is then processed using the Pandas library, which efficiently parses the CSV data into a data frame for subsequent processing. Although the full dataset is loaded into memory, this approach is optimized for the typical study sizes encountered in clinical research settings. The extracted data is then written to disk in CSV format alongside export metadata.

The connector includes error handling for HTTP response codes, malformed metadata and authentication failures. Detailed diagnostic output supports fault investigation, while API tokens are handled according to institutional data-protection and information-security procedures.

#### Extensibility benefits

The Strategy pattern enables runtime selection of data sources without altering core code, supporting independent development and testing of connectors. It maintains backward compatibility and enforces a standardized interface across all connectors, simplifying integration. This design also facilitates isolated unit testing and allows easy extension to future data sources like FHIR, XML, and databases.

**Code snippet 4** Future Connector Examples

**Figure:**
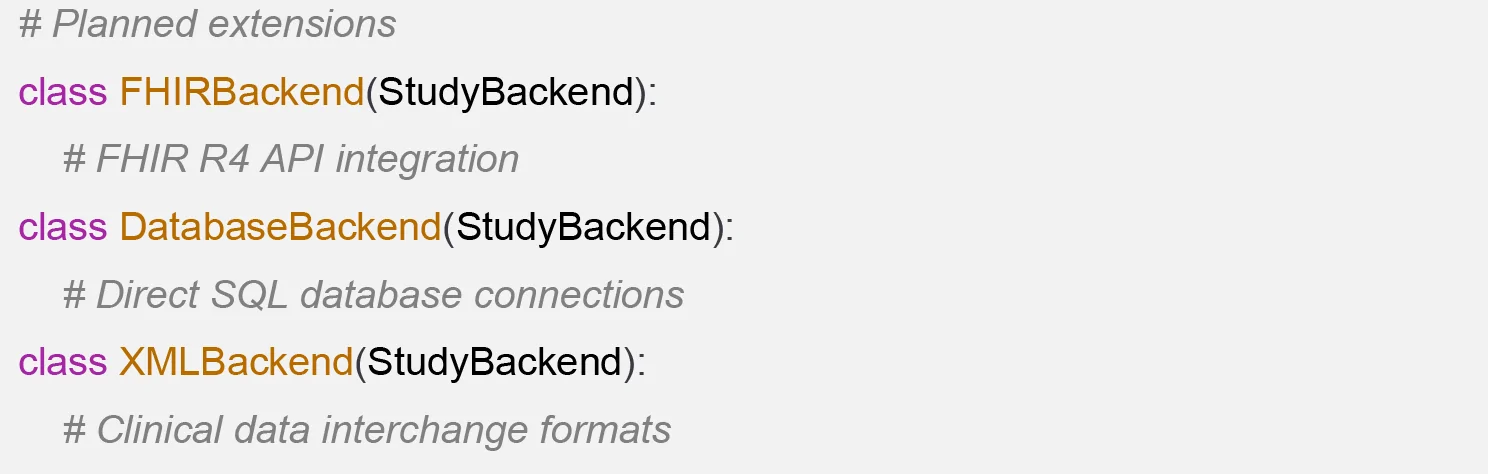

**Fig. 2:**
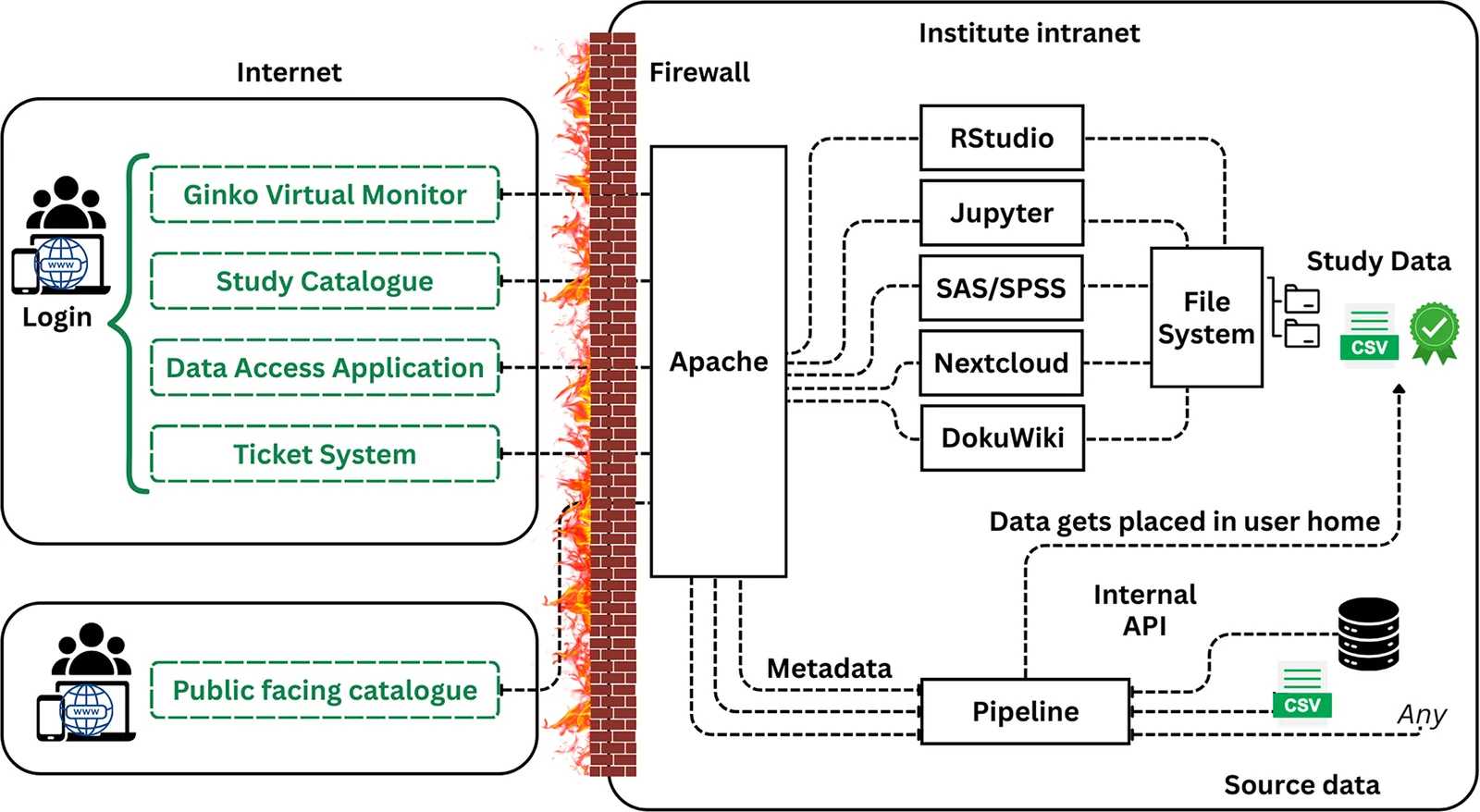
Trusted research environment at our institution. The diagram illustrates the separation between public-facing internet components (left) and the secure institutional intranet (right), mediated by a firewall and Apache web server. Authenticated users access the Ginko Virtual Monitor, Study Catalogue, Data Access Application, and Ticket System through secure login, while the public-facing catalogue remains accessible without authentication. Within the institute intranet, approved researchers work with analytical tools (RStudio, Jupyter Notebook, and the commercial statistical analysis software packages SAS and SPSS), collaboration platforms (Nextcloud), and documentation systems (DokuWiki), all connected to a centralized file system containing verified study data. The data pipeline extracts approved data through internal APIs and provisions the resulting datasets to users’ home directories on the internal file server for access through Ginko

The connector architecture is designed to integrate additional data sources without changes to the TRACE core, although connector-specific development and configuration may be required; REDCap and CSV connectors are currently implemented. Figure 2 summarises the overall system architecture.

### Secure data access and workflow management process

Sensitive tools and data, including REDCap, RStudio, Nextcloud and Jupyter Notebook, remain securely isolated within the institutional firewall. Researchers without institutional VPN access analyse datasets through the secure VNC-based Ginko Virtual Monitor, which provides controlled, intranet-only access to analytical tools. This architecture separates public metadata exploration from authenticated access to analytical tools and study data within the institutional intranet.

Once a data request has been approved, a cron-based shell script using SSH automatically provisions the approved extract to the researcher’s home directory on an internal file server and to a separate archive directory. Researchers without institutional VPN access analyse TRACE-provisioned extracts through the Ginko environment, where direct file downloads and clipboard transfer to local machines are disabled. Authorised internal staff and study-team members may retain their existing access to the source system through the institutional intranet or VPN and may export data from that system where the data owner approves. TRACE is intended to provide an additional governed access pathway rather than to replace all existing authorised internal access routes.

Users can browse the TRACE study catalogue online to search for studies of interest. Once a researcher has successfully applied for data access via a formal application through TRACE, they can access the TRE (the intranet) via the Ginko Virtual Monitor and begin working with the data. The study variable selection initiated by the researcher is persistently saved and linked to the study, enabling this selection to be easily reused in future projects. Therefore, researchers can browse studies and their sub-projects and apply for data sets that have been requested in the past.

#### FAIR principles and auditability

The platform supports Findable, Accessible, Interoperable and Reusable (FAIR) data by providing a transparent, auditable and structured workflow for accessing study information and requesting data. As a locally hosted solution, TRACE allows institutions to establish and apply standards of their choice across all studies, capturing and presenting this standardised metadata to facilitate consistent, interoperable data sharing. The extent to which TRACE meets the FAIR Maturity Indicators is assessed in the Results (Additional File 2).

#### Alignment with the TRUST principles

Leavitt’s Diamond is a foundational framework for organisational change which shows that people, tasks (processes), technology and organisational structure are tightly interdependent [21]. If one of these elements changes, adjustments must be made to the others for the organization to remain effective. In the context of data sharing, introducing TRACE as a technological innovation drives changes in researcher practices, governance workflows and institutional oversight structures. Lin et al. articulate expectations for trustworthy digital repositories through the community-endorsed TRUST Principles, which emphasize transparency, responsibility, user focus, sustainability, and technology [22].

#### Transparency

TRACE records data access requests, approval decisions and dataset extractions, supporting traceability of these workflow events. Study metadata, governance information and applicable data-usage agreements can also be documented through the platform.

#### Responsibility

TRACE supports GDPR-related handling through access control, audit logging and data minimisation, preserves dataset authenticity through version freezing, and records provenance for each approved extract. Compliance itself remains a legal and organisational responsibility of the data controller, which the platform documents but cannot enforce.

#### User focus

Researcher-driven variable selection, metadata-rich catalogues, and reusable sub-projects are designed to serve the research community’s needs directly.

#### Sustainability

TRACE is open source, institutionally supported, and maintained via structured Git workflows; archival policies are intended to support long-term viability, maintainability, and extensibility.

#### Technology

The technical stack combines Django, LDAP authentication, a modular connector architecture and analytical tools such as RStudio and Jupyter Notebook within the Ginko environment to support controlled access to sensitive data.

Together, these features align TRACE with several elements of the TRUST principles and are intended to support long-term, reproducible, and responsible data sharing.

### Testing and deployment

The TRACE platform development follows a structured Git workflow via the institutional GitLab environment (Leibniz-Rechenzentrum (LRZ) GitLab). Features are developed, tested locally (e.g., using Jupyter Notebook for prototyping), reviewed, and progressively deployed through clearly defined branches (development → production → main). This workflow supports traceability of code changes and controlled deployment of reviewed updates to the production environment.

### Data security

The current TRACE implementation includes several technical and organisational controls. Authentication is provided through institutional LDAP, while authorisation is managed through Django’s role-based permissions. Web traffic is protected using TLS. For researchers using Ginko, direct file downloads and clipboard transfer to local machines are disabled. Each relevant relational model has an associated history table that records insertions, updates and deletions together with available user and timestamp information. These controls are intended to support access restriction, traceability and activity monitoring; their effectiveness in reducing security incidents has not been formally evaluated.

### Alignment with the five safes framework

TRACE was designed with reference to the widely used Five Safes framework for data access [23]. The following paragraphs map TRACE’s current controls and limitations to the framework’s five dimensions. Each dimension of the framework is addressed through specific technical and organisational controls implemented in TRACE, designed to support responsible and secure use of sensitive research data within institutional legal and governance processes.

#### Safe projects

All data access requests are submitted through a TRACE workflow. Each project undergoes ethical and legal review, and a data usage agreement must be signed before approval.

#### Safe People

Access is restricted to authenticated institutional users via LDAP. Role-based permissions and model-change history through django-simple-history support accountability and traceability.

#### Safe data

Only pseudonymised and pre-approved data are made accessible. Variable-level selection limits each extract to fields approved by the data owner.

#### Safe settings

Researchers without institutional VPN access analyse TRACE-provisioned extracts through Ginko, where direct file downloads and clipboard transfer to local machines are disabled. Authorised internal staff and study-team members may instead access the source system through the institutional intranet or VPN where the data owner approves.

#### Safe outputs

Outputs remain within the Ginko environment until they have been reviewed manually by the data owner. An integrated disclosure-review and monitored-export workflow has not yet been implemented and is included in the planned development roadmap (Table 4).

## Results and discussion

This section reports observed usage of TRACE, describes its core functionality, positions it within the TRE landscape, and discusses limitations and future development. The results reported here are descriptive metrics from a single-site deployment; improvements in efficiency, security and error rates were not directly measured and are discussed as expected, rather than demonstrated, benefits.

### Observed usage and changes in export workflows

We analysed REDCap event logs for the three studies onboarded to TRACE (PEACHES, BEARR and ENABLE; four REDCap projects across two instances, of which the BEARR randomization project was excluded). Between 1 January 2022 and 18 March 2025, the date of TRACE go-live, 1,655 manual, interface-initiated data exports were recorded, corresponding to 42.4 per 30 days. Between 18 March and 31 December 2025, 45 such exports were recorded, corresponding to 4.7 per 30 days — a decrease of 89.0%. Manual exports did not cease, and were not intended to: 45 were recorded after go-live, chiefly by data-management staff, for whom direct access to the source system with data-owner approval remains the intended route. These are uncontrolled before–after observations derived from routine log data. The decrease is temporally associated with the introduction of TRACE, but the study design does not support causal attribution: export volume also varies with study lifecycle stage independently of platform change. Export counts record the number of occasions on which an export action was processed by the source system; they are not a direct measure of data exposure, administrative effort or error rates, and improvements in those outcomes are inferred rather than demonstrated. Full derivation, including definitions, inclusion criteria and queries, is provided in Additional File 4.

The request workflow changed from a manual, email-based process to a self-service process within the platform. As of December 2025, seven studies had been implemented in TRACE, 17 researchers were registered as active users, and 56 data access applications had been processed. These counts describe uptake during the initial deployment period. They were not accompanied by measures of user effort, task completion time, or error rates, so they document use of the platform rather than an improvement in the work it supports.

#### Context

The surrounding REDCap infrastructure. TRACE is deployed alongside our institution’s existing REDCap installation, which comprises 298 active study centres across 21 multicentre studies and more than 1,100 registered users (Table 1). These figures describe the potential user base within the institution’s existing collaborative network; they do not represent TRACE adoption. During the observation period reported here, TRACE was used across seven studies by 17 registered researchers and processed 56 data access applications. Whether TRACE can scale to a population of this size has not been evaluated, and no load or performance testing at that scale has been performed.

**Table 1:** Context and observed usage. Rows labelled “Institutional REDCap” describe the surrounding infrastructure and the potential user base; rows labelled “TRACE” describe observed usage as of December 2025

Metric	Value	System
Registered users	+ 1,100	Institutional REDCap (context)
Study centers	298	Institutional REDCap (context)
Multicenter studies	21	Institutional REDCap (context)
TRACE registered studies	7	TRACE (observed usage)
Active researchers	17	TRACE (observed usage)
Data access applications	56	TRACE (observed usage)

The network includes more than 25 affiliated centres in North America and Europe. These figures describe the reach of the existing REDCap collaborations rather than of TRACE: of the 17 researchers registered to date, 15 are based at our own institution and two at other German universities.

These results describe observed changes in export activity and platform usage during the initial deployment period. The following sections address core functionality, the analysis environment and comparative benchmarking.

### Core functionality and workflow design

**Fig. 3:**
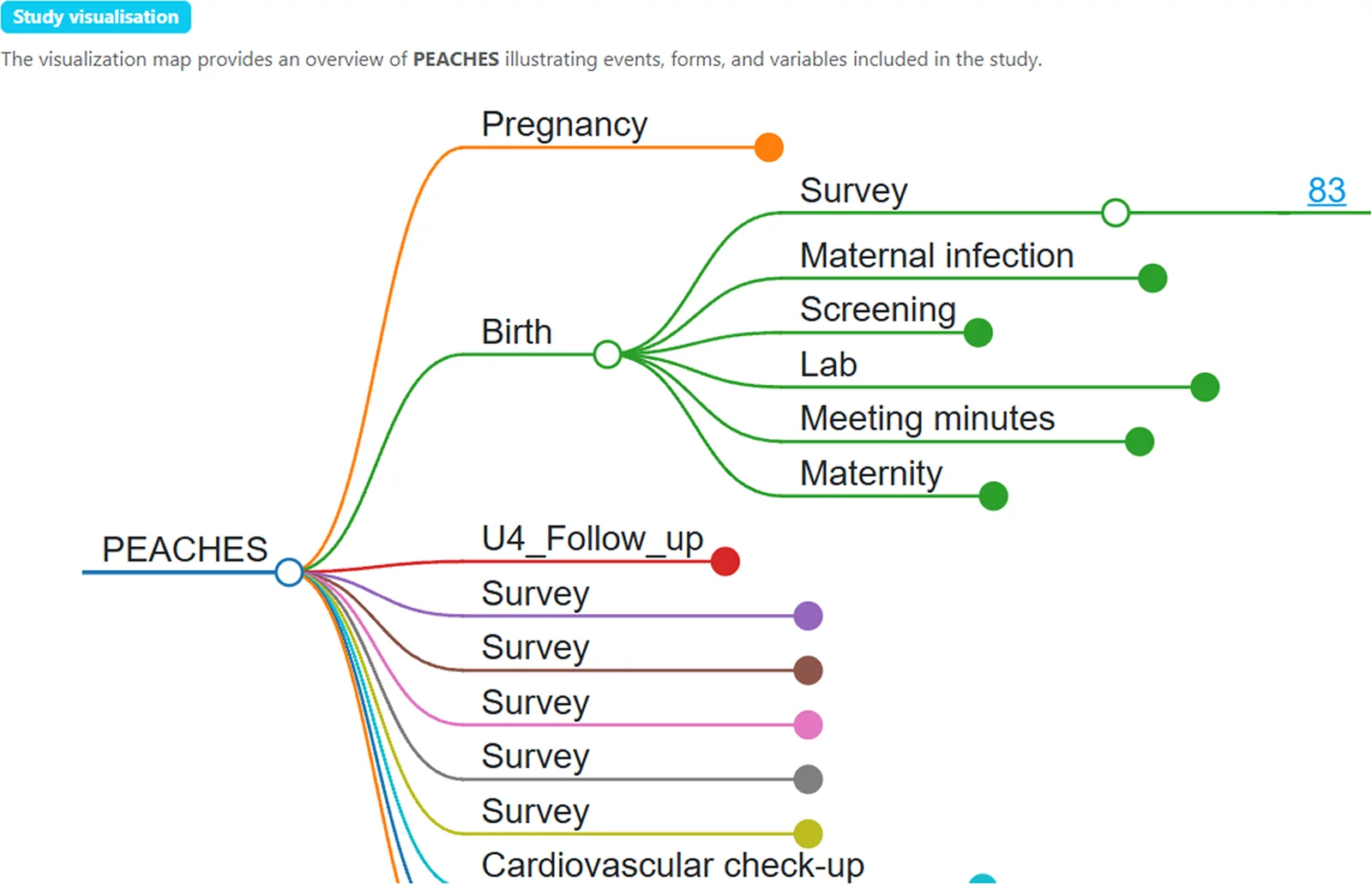
TRACE interactive metadata explorer interface. Users navigate a hierarchical structure of metadata (Study title → Event → Instrument → Variable count). In this example, the user explores the metadata hierarchy of the PEACHES study: PEACHES → Birth → Survey at Year 0 → 83 variables

**Fig. 4:**
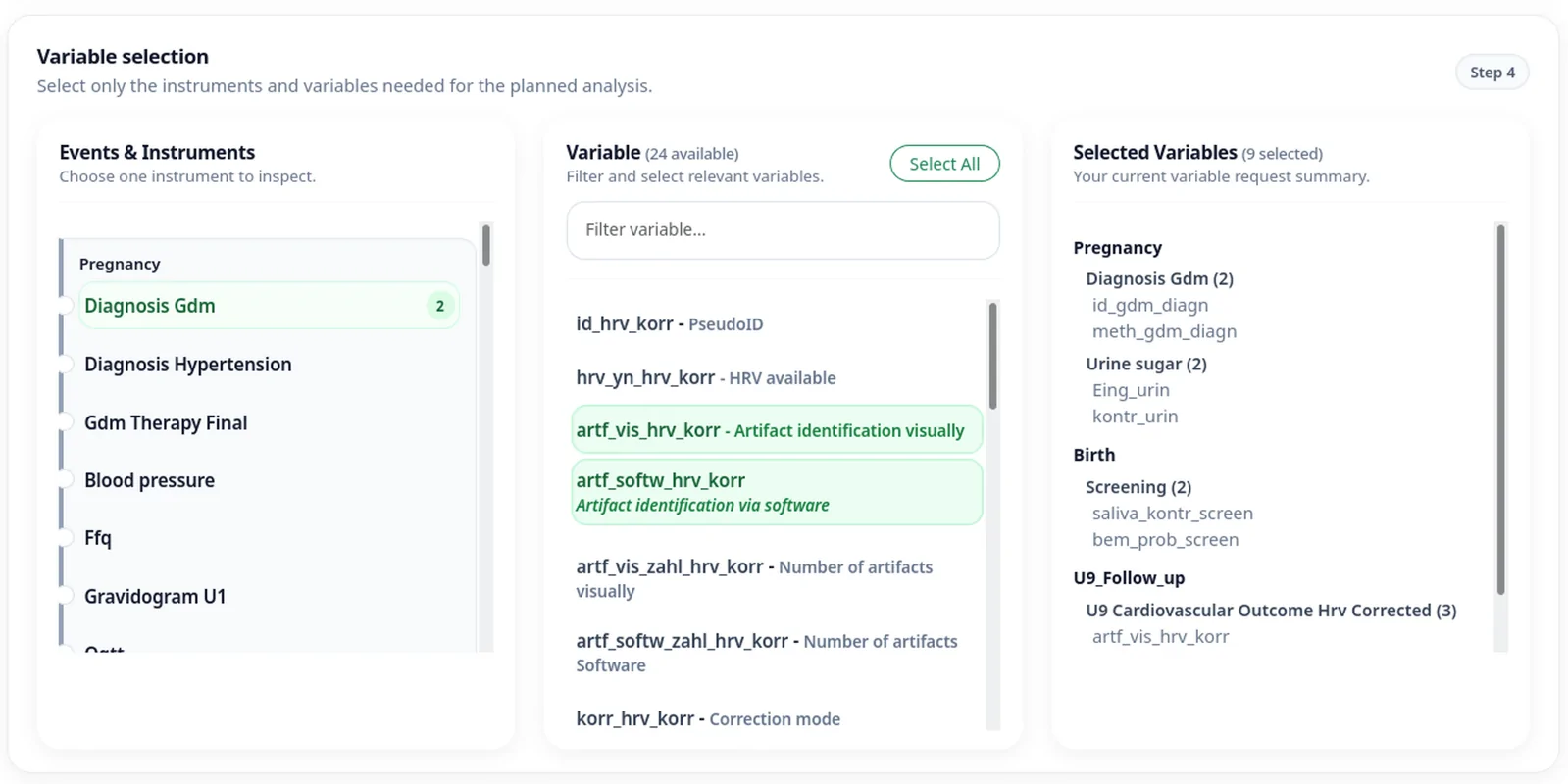
TRACE variable-selection interface. This screenshot demonstrates the researcher-driven selection of individual variables from the 83 available within the instrument “Survey at Year 0” associated with the “Birth” event of the PEACHES study

A central feature of TRACE is its interactive variable selection interface, which allows researchers to explore data sources and select only the relevant variables for their data requests via the web application (see Figs. 3 and 4).

While some platforms provision complete study datasets into secure workspaces, TRACE supports graduated disclosure. For data owners it acts as a valve in two dimensions: which variables leave the study, and the setting in which the recipient works with them.

The first dimension is implemented in the platform. Data owners authorise extracts that are narrowly scoped and aligned with protocol and consent constraints, which reduces the perceived loss of control while enabling reuse. The second dimension is governed organisationally: researchers without institutional VPN access use the Ginko route for approved extracts, whereas authorised internal staff and study-team members may retain access to the source system through the institutional intranet or VPN. The applicable route is determined jointly by the data owner and the TRACE team.

The interface dynamically adapts to both longitudinal and cross-sectional project structures, loading available instruments and variables using structured metadata retrieved from the source. Researchers’ selections are displayed in real time and saved in a structured JSON format, which supports downstream data extraction and analysis.

The interface is implemented with JavaScript on the front end and Django templates for context-aware rendering on the back end. It is intended to reduce administrative effort and to make the data access process more transparent, since the variables requested are recorded explicitly with each application. Because approved extracts are provisioned inside the sealed environment, the workflow removes the routine need to move data out of the source system manually. TRACE also introduces centralised workflow traceability through integrated logging and a central tracking system, replacing disjointed email threads with a centrally logged and traceable workflow.

Table 2 contrasts the legacy workflow with the workflow that TRACE is designed to support. The entries describe design characteristics and intended changes in process; the criteria were not measured before and after implementation, and the table is not the result of a comparative evaluation.

**Table 2:** Design comparison of the legacy workflow and the TRACE-enabled workflow

Criterion	Legacy Workflow	TRACE Workflow
Access route	Manual, email-based request; no structured record of what was requested and why	Self-service web interface with a structured application form, and links to the study and application in notification emails
Security	Data leaves institutional research platform, manual cloud transfer	TRACE-provisioned extracts are accessed through Ginko without direct file downloads or clipboard transfer; authorised internal staff may separately access the source system through the institutional intranet or VPN with data-owner approval.
Process structure	Manual, multi-step, dependent on individual coordination	Structured workflow with automated provisioning and central tracking
Traceability	No consistent audit trail	Central workflow records and model-change history through Django and django-simple-history.
Metadata Access	Requires direct contact with staff	Publicly browsable structured study metadata in TRACE.
Support Workflow	Unstructured email threads	Centralized ticketing system for questions to the admins
Results Access	Not controlled or specified	Manual data-owner review before release; integrated disclosure-review and monitored-export workflow not yet implemented.

Entries describe intended process characteristics, not measured outcomes

#### Enhanced reusability through public sub-projects

**Fig. 5:**
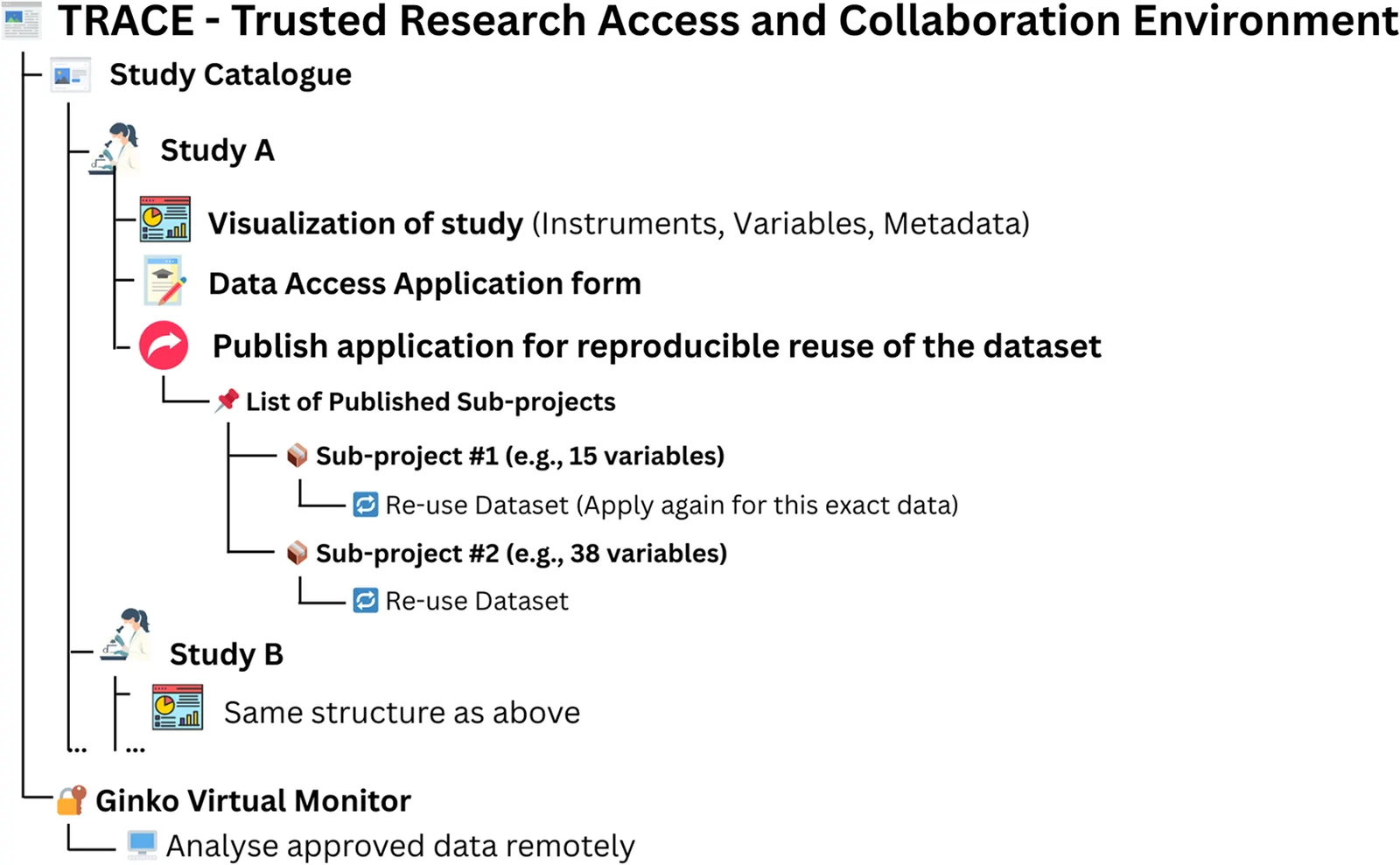
Conceptual mock-up of the TRACE platform’s public-facing sub-project listing. Each sub-project (e.g., “Sub-project #1”, “Sub-project #2”) represents a previously approved data-access application. Researchers can reference a static, versioned dataset (CSV) along with metadata and optionally share analysis scripts (R, Python), enabling re-analysis of the identical dataset with the original scripts despite subsequent updates to the main project

TRACE supports the conversion of approved data access applications into publicly listed sub-projects. Once an application has been approved, researchers can choose to publish their selected dataset (‘frozen’ as a static CSV export) alongside the relevant metadata directly onto the publicly accessible study page. This allows future applicants requesting access to the same sub-project to receive the archived dataset version, even if the main study subsequently changes. Original analysis scripts in R and Python can also be shared, thereby enhancing reproducibility and the potential for reanalysis (see Fig. 5).

TRACE therefore supports reproducibility at the level of data and analysis code: a later applicant receives the identical frozen dataset together with the scripts and can re-run the analysis inside the TRE. The computational environment is not part of the snapshot — package versions are not pinned, no container images are stored, and the analysis virtual machines are non-persistent — so identical numerical results across time or across sites are not guaranteed. Capturing the environment, for example through renv or pip freeze manifests or a container image per sub-project, is planned (see Future Work).

These capabilities are relevant to the platform’s suitability for complex study onboarding. The value of these features is illustrated by our experience of onboarding the PEACHES study. Migrating the study from Microsoft Access to REDCap required us to harmonize over 2,400 variables, many of which had previously been defined only as labels or cryptic codes scattered across various formats. We enabled structured metadata access and transparent selection for downstream analysis by building a structured codebook, enriching metadata in REDCap with the Custom Codebook external module, and leveraging TRACE’s automated variable ingestion. By contrast, the ENABLE and BEARR studies, which already used richer native REDCap metadata, only required minor adjustments to be made, describing TRACE’s flexibility with regard to diverse study origins and metadata formats.

These examples describe how studies with different metadata quality were onboarded, and suggest that the connector approach accommodates both. They illustrate the intended workflow rather than measured usability, which has not been formally evaluated.

### Ginko virtual monitor

**Fig. 6:**
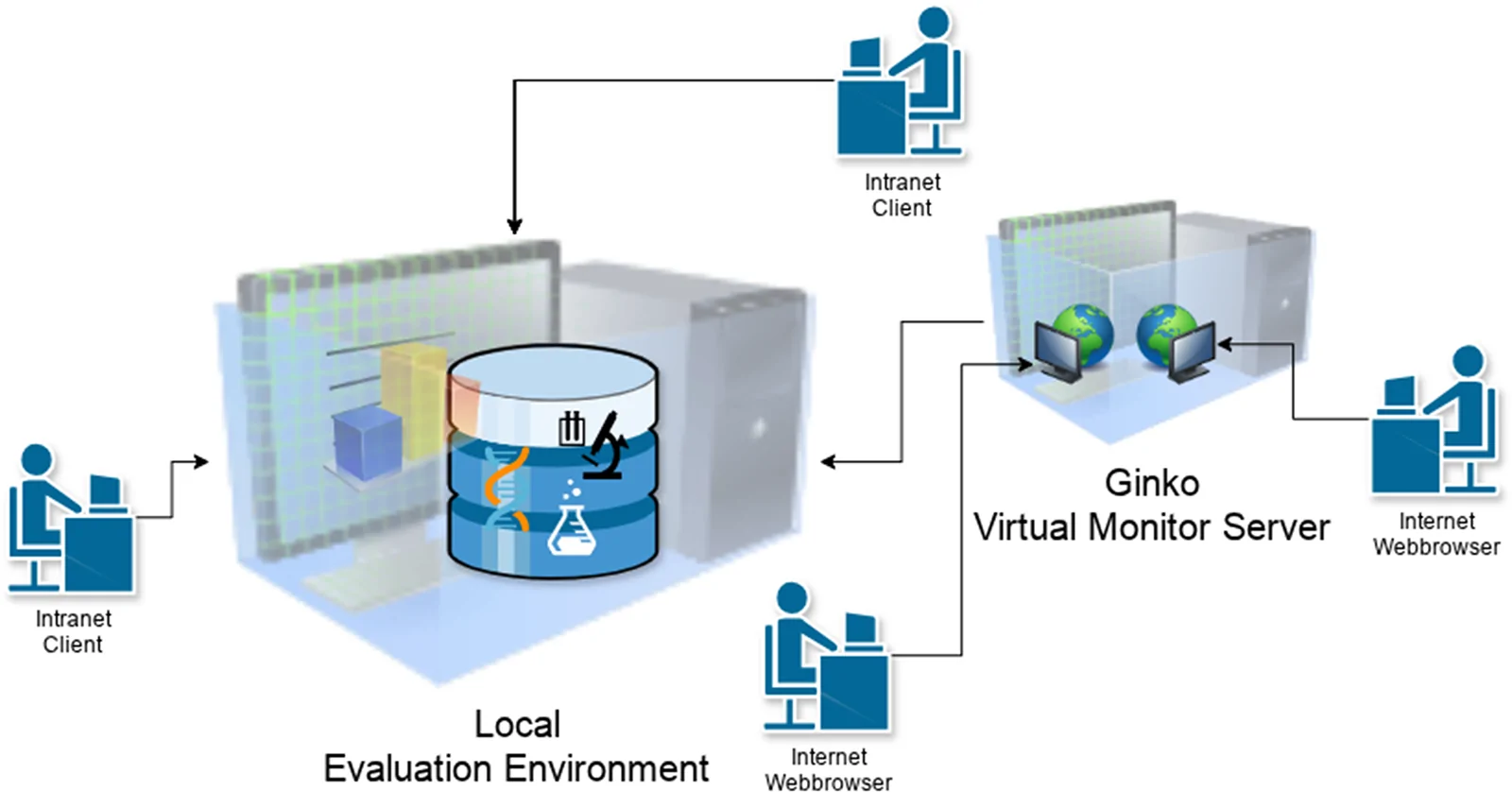
Ginko virtual monitor. Authenticated researchers access analytical tools (RStudio, Jupyter, Nextcloud) through a browser-based VNC session. The sealed environment disables direct file downloads and clipboard transfer to the user’s local machine while permitting copy-and-paste operations between applications within the institutional intranet

Ginko Virtual Monitor provides registered users with browser-based access to tools hosted within the institutional intranet through non-persistent virtual machines (Fig. 6).

Copy-and-paste remains possible between applications within the institutional intranet, for example from RStudio to Jupyter Notebook or ONLYOFFICE; screenshot-based exfiltration remains a residual risk. Technically, Ginko is based on QEMU (quick emulator)-Web-Desktop (QWD), a web application that allows authenticated users to create and select non-persistent QEMU-KVMs (KVM, kernel-based virtual machines) that are accessible via a browser and have a limited lifetime.

For the Ginko deployment, QWD was modified to block multiple simultaneous connections to one virtual machine, restrict network access through host-based rules, and support maintenance of template virtual machines using libvirt and virt-manager.

The custom template VMs have a very limited user interface only allowing configuration of screen resolution, keyboard layout and accessing our data processing environment using Firefox. No other websites are accessible.

### Benchmarking and Positioning In the TRE landscape

We compared the capabilities of TRACE with those of well-known existing solutions, such as Vivli, HONIC, OpenSAFELY and YODA (Yale Open Data Access) [24–27].

These existing platforms differ considerably in terms of openness, deployment model, data management, researcher autonomy and compliance frameworks. TRACE provides a flexible, open-source, locally hosted approach that allows institutions to retain local control over sensitive datasets and is designed to support compliance with applicable data-protection requirements, particularly the General Data Protection Regulation.

Table 3 provides a concise summary comparing the key features of the TRACE platform with those of selected existing platforms.

**Table 3:** Comparative feature analysis of TRACE and selected data-access and secure-analysis platforms

Feature	TRACE	Vivli	HONIC	OpenSAFELY	Yale YODA
Software/ service model	Open-source software	Hosted non-profit data-sharing platform	Commercial hosted data service	Open-source analytics platform	Academically governed data-access service
Hosting and data locality	Institutionally hosted; institution retains local data residency	Cloud-hosted Vivli platform; governed access involving Vivli and the data contributor.	Honic-hosted German/EU platform; project access occurs within Honic’s controlled analysis environment.	In-situ deployment within NHS EHR-provider data centres; controller roles include GP practices and NHS England depending on the data layer.	Secure platform or secure electronic transfer, depending on the data partner
Primary data focus	Source-agnostic clinical research data; REDCap and CSV connectors implemented	Clinical research data, particularly completed interventional studies; some observational studies	German real-world care data from participating healthcare providers.	NHS EHR data, particularly primary-care and linked records	Clinical-trial data contributed by participating data partners
Cost information	No software licence fee; deploying institution bears infrastructure, staffing and security costs	Data request free; secure-environment charges may apply depending on duration and capacity	Public price schedule not identified; consultation required	Annual NHS England service licence fee of £25,000 excluding VAT for project teams as of 2026; access restricted to approved projects.	Data provided free of charge; approved scientific, non-commercial use only
Request and selection granularity	Researcher-driven variable-level selection through the interface	Study/data-package request; self-service variable-level selection not publicly documented	Project-specific cohort and data package prepared with Honic; self-service variable-level selection not documented	Programmatic cohort and variable selection via ehrQL	Proposal-scoped data release; self-service variable-level selection not documented

Sources: Vivli [24], Honic [25], OpenSAFELY [26, 28–30], and Yale YODA [27, 31]. Platform information reflects publicly available sources reviewed on 2 August 2026. “Not publicly documented” means that the feature was not identified in the sources reviewed and should not be interpreted as evidence that the feature is absent

Vivli provides a cloud-hosted platform for governed access to clinical research data, whereas the YODA Project uses an academically governed trusted-intermediary model to review proposals and enable access to clinical-trial data contributed by participating data partners [24, 27]. Honic provides project-specific access to German real-world care data within a controlled analysis environment hosted in the European Union [25]. OpenSAFELY differs from these hosted access services by enabling analyses to run in situ within NHS electronic health record provider data centres, thereby avoiding the transfer of large volumes of potentially disclosive pseudonymised patient data off-site [26]. In this selected comparison, TRACE complements these approaches through local institutional deployment, researcher-facing variable-level selection, connector-based metadata integration, versioned dataset extracts, and institutional control of the access workflow.

#### FAIR principles benchmarking

As a structured self-assessment, we scored TRACE against the FAIR (Findable, Accessible, Interoperable, Reusable) Maturity Indicators, which are endorsed by the community [32]. Each indicator was systematically scored to measure the extent to which our platform adheres to international standards, as shown in Additional File 2.

In this structured self-assessment, TRACE met most of the assessed essential indicators, while globally registered identifiers and several data- and community-standard-related elements remain incomplete or dependent on study configuration (Additional File 2). Additionally, TRACE now provides structured, machine-readable metadata encoded in JSON for linked data (JSON-LD) that aligns closely with schema.org standards for datasets (see Additional File 3). An example from the PEACHES study illustrates the level of detail in the captured metadata, including variable-level descriptions and standardised identifiers. These implemented features close the machine-readable-metadata gap from the earlier self-assessment, and study- and variable-level metadata now carry stable TRACE URIs (F1-01 M). Globally registered persistent identifiers (DOIs) for studies and dataset extracts remain planned (see below). Equivalent coverage for other connector types remains under development, and our future plans include the explicit mapping of metadata elements to widely recognised biomedical ontologies (e.g. SNOMED CT (Systematized Nomenclature of Medicine Clinical Terms), ICD (International Classification of Diseases), LOINC (Logical Observation Identifiers Names and Codes)) to further enhance interoperability and alignment with FAIR principles. One of the next steps is to integrate LMU library DOI services to provide a persistent identifier for each study in the catalogue for publication sharing.

### Educational impact and support for open science training

Following the recent launch of CONSORT 2025 and SPIRIT 2025, there has been a significant push towards open science practices. SPIRIT (Standard Protocol Items: Recommendations for Interventional Trials) provides guidance on how to write a clear and complete trial protocol. CONSORT (Consolidated Standards of Reporting Trials) provides guidance on transparently reporting the results of a completed trial. The ‘Open Science’ element introduced in both represents a significant development, bringing clinical trials into line with the wider push for research transparency, accessibility and reproducibility. In SPIRIT 2025 and CONSORT 2025, open science refers to the structured and prospective declaration in protocols and transparent reporting in results of the following: Data Sharing Plans, Access to Trial Materials, Registration and Dissemination. We train students to implement studies within TRACE so that they learn how to prepare data for sharing and comply with FAIR principles. They are introduced to a system that will facilitate their participation in clinical trial data sharing later in their careers. Conversely, we provide doctoral students with access to data via TRACE, offering training in best practices for using shared data. This familiarizes them with practices that will shape their ideas of transparency and collaboration.

The European Health Data Space Regulation establishes a framework for the secondary use of electronic health data, including access authorisation, data minimisation and processing in secure environments [33, 34]. TRACE has not been evaluated for conformity with the Regulation. Its structured application workflow, variable-level selection, audit logging and Ginko-based restricted-analysis pathway relate to several operational elements addressed by the Regulation [33, 35]. The applicability of specific EHDS requirements to TRACE’s different access routes, as well as the relevant implementing acts and national arrangements, remains to be assessed [34, 36].

### Limitations

To provide transparency regarding the current state of TRACE and its planned evolution, Table 4 summarizes the security roadmap, differentiating between implemented features, short-term development goals and requirements for a production-ready institutional deployment.

**Table 4:** Roadmap for security and trustworthiness in TRACE, from prototype to hardened institutional deployment

Phase	Scope & Focus	Security / Trust Measures
Prototype (current)	Demonstrator implementing FAIR-aligned metadata workflows and a researcher-facing access interface.	· Role-based permissions · TLS for data in transit · Database audit logging · Controlled access to pseudonymized datasets via VNC (no direct downloads)
Next steps (planned)	Harden the released open-source platform for users beyond the home institution: external accounts, documented deployment.	· Formalized threat model (assets, adversaries, risks) · Two-factor authentication for sensitive actions · Key management and rotation practices · Initial result-exfiltration controls (monitored export) · Deployment and process documentation
Production-ready / Hardened	Production-level TRACE suitable for broad institutional adoption.	· Comprehensive STRIDE/LINDDUN threat analysis · Penetration testing and “red teaming” · Incident response and recovery plan · Tamper-evident, long-term audit chain (hash-chained logs / WORM storage) · Continuous monitoring and regular security audits

This report describes a single-site implementation with an uncontrolled before-and-after comparison of routine log data. No concurrent control group or experimental design was used, no outcome was tested against a predefined hypothesis, and no causal conclusions can be drawn. The export figures are temporally, but not causally, associated with the introduction of TRACE, and export volume may also vary with the lifecycle stage of a study independently of any platform change.

#### Single site

TRACE was deployed at one academic medical centre, with one dominant source system (REDCap), one legal framework and one governance culture. Institutions with different source systems, different data protection advice or different staffing may reach different conclusions about the effort required; generalisability beyond this setting is untested.

#### Scale

Observed usage covers seven studies, 17 registered researchers and 56 data access applications during the first months of operation. These numbers are too small to characterise behaviour under load, to detect rare failure modes, or to support claims about scalability.

#### Selection

Studies and users were not sampled. The studies onboarded first were those whose data owners were already in contact with our institute and were willing to adopt a new workflow. Of the 17 registered researchers, 15 are institute-affiliated and two are based at other German universities. Institute-affiliated users are likely to be more favourably disposed towards the platform, and more tolerant of its rough edges, than unaffiliated external applicants would be, so the early experience reported here is not representative of external use.

#### No user-centered evaluation

Ease of use was a design objective, but no formal user-centered evaluation—for example System Usability Scale scores, task-based testing or structured interviews—has been performed. The usability statements in this manuscript reflect design intent and informal feedback from early users.

#### No direct outcome measures

We did not measure the time required to fulfil a data request, the number of data handling errors, staff effort, or security incidents, either before or after implementation. Statements about efficiency, error reduction and security are inferred from properties of the workflow—auditability, automated provisioning, analysis inside a sealed environment—rather than demonstrated by measurement.

#### Implication

Taken together, these constraints mean that the present findings support feasibility—that a platform of this kind can be built and operated by existing institutional staff—but not effectiveness. Comparative, multi-site and user-centered evaluation would be needed before an effect on efficiency, error rates or data protection outcomes could be claimed.

#### Limitations of the platform’s current scope

Firstly, it should be noted that TRACE is not designed to replace dedicated data management platforms such as REDCap or institutional registries, as its scope presupposes upstream data curation and collection. However, it is intended to integrate seamlessly with such systems, and formal integration guidelines will be developed and disseminated.

Secondly, the current user management system relies on institutional LDAP and Django’s internal authentication framework, which limits flexibility for external collaborators. Nevertheless, a secure external account system featuring password recovery, two-factor authentication and self-service management is planned to enhance accessibility while ensuring institutional oversight.

Finally, TRACE does not enforce domain-specific study design standards, as responsibility for methodological rigour remains with the principal investigator and study team. Nevertheless, the platform facilitates transparent communication of study structures and metadata, thereby promoting sound data governance, interoperability, and responsible data sharing.

## Future work

Future development will prioritise scalability, maintainability, performance and user experience. Firstly, database scalability will be addressed by migrating from SQLite to PostgreSQL. This is expected to provide production-grade performance, transactional integrity and efficient support for complex queries, while maintaining compatibility with components such as Ginko, Nextcloud and analysis environments. Secondly, front-end modularisation and the adoption of a modern framework (e.g. React) are intended to improve maintainability, developer productivity and the user experience. Thirdly, caching and API rate-limiting mechanisms will be implemented to optimise metadata retrieval and system responsiveness without compromising data freshness.

### Further planned developments include

- Automated publication integration via APIs such as CrossRef, Europe PMC, and PubMed to enhance study discoverability.
- Systematic security hardening through penetration testing, code auditing, and targeted training.
- Comprehensive onboarding materials to facilitate institutional adoption.
- Evaluation of AI-assisted system monitoring and local AI-supported research workflows for potential use within environments such as RStudio and Jupyter Notebook.
- A structured usability evaluation of the researcher-facing workflow.
- Capture of the computational environment for published sub-projects (renv and pip freeze manifests, and container images, for instance, where feasible) so that a frozen dataset and its scripts can be re-run in a defined software environment.

The current TRACE core does not include AI functionality evaluated in this study. Before planned AI-assisted monitoring or research components are deployed, their status under the EU AI Act should be assessed according to their intended purpose, applicable risk classification and the institution’s role as provider or deployer.

## Conclusions

This work describes an implementation in which lightweight, open-source infrastructure was deployed to support FAIR-aligned data sharing within a single institution. In our implementation, TRACE processed data-access requests with central workflow logging and maintained versioned dataset snapshots, allowing the same data and analysis scripts to be re-analysed within the environment. It also provided sealed analysis environments to control access. TRACE replaced part of the previous informal exchange and manual export of data; manual exports from the source system decreased but did not cease. Three practical insights emerged. Firstly, TRACE hides the differences between various data sources by treating them all through the same ‘connector’ model. Even though studies may originate from REDCap, CSV files or, in the future, FHIR or database systems, each source is converted into a common internal format. Secondly, researcher-driven variable selection enables narrowly scoped data extracts that are aligned with consent and protocol requirements. This enables narrower extracts than full-study exports and makes the requested variables explicit to data owners; the actual change in exported data volume was not measured. Thirdly, in this implementation, integrating metadata cataloguing, access control and analysis environments into a single platform allowed existing institutional staff to manage requests without establishing a dedicated team. This suggests, but does not demonstrate, that the approach is feasible for settings with limited resources.

Compared with other components of the data-sharing ecosystem, TRACE provides a complementary option for institutions that must retain local control over sensitive data. In the selected comparison presented in Table 3, TRACE differed by combining local institutional deployment, researcher-facing variable-level selection, versioned dataset extracts, and an integrated controlled analysis pathway within one institutional workflow. Instead, TRACE prioritises local hosting, versioned extracts and structured governance, making it a potentially relevant option for institutions operating under GDPR and for research groups requiring reproducible access to clinical or operational data. Suitability and implementation requirements in other institutional settings remain to be evaluated.

Several developments will strengthen production readiness. Short-term priorities include implementing two-factor authentication for external users, conducting formal threat modelling (risk analysis) and improving the variable-selection interface. Medium-term goals include penetration testing, a disclosure-review workflow for the controlled export of analysis results, the integration of federated identities (e.g. logging in with ORCID) and refactoring the front end for maintainability. Longer-term plans involve integrating a local code assistant within sealed environments, automating the linkage of published outputs via CrossRef/PubMed APIs and including terminologies such as SNOMED CT and LOINC to support interoperability. In parallel, standard operating procedures, deployment templates and multi-site pilot evaluations will be developed to assess scalability across institutional contexts. In our single-site implementation, TRACE was deployed using existing institutional infrastructure and staff; requirements at other institutions have not been evaluated.

### Availability and requirements

Project name: TRACE - A Trusted Research Access & Collaboration Environment.

Project home page: https://trace.ibe.med.uni-muenchen.de - code available via https://gitlab.lrz.de/trace.

Operating system(s): Linux server deployment (developed and tested on Debian GNU/Linux); client access via any modern web browser.

Programming language: Python, JavaScript.

Other requirements: Python 3.13 with Pandas library, CSV parsing and JSON handling library, Django 5.2.11, SQLite, LDAP, TLS/HTTPS, REDCap API client, Ginko Virtual Monitor, QEMU-KVM, libvirt, virt-manager, Nextcloud, RStudio, Jupyter Notebook, web browser.

License: GNU Affero General Public License version 3 or later (AGPL-3.0-or-later).

Restrictions on use by non-academics: None based on user type or commercial status. Use, modification and redistribution are subject to the AGPL. Where a modified version is offered as a network service, users interacting with it must be offered access to the corresponding source code under the licence terms.

## Electronic Supplementary Material

Below is the link to the electronic supplementary material.

Supplementary Material 1

## Data Availability

We share our work on GitLab - no real study data but all other data relevant to this work. For testing the application without a login: Link to TRACE: https://trace.ibe.med.uni-muenchen.de/. Link to study catalogue: https://trace.ibe.med.uni-muenchen.de/projects/. Link to PEACHES study: https://trace.ibe.med.uni-muenchen.de/harvest/project/1/. Link to PEACHES JSON-LD: https://trace.ibe.med.uni-muenchen.de/harvest/project/1/metadata.json/. Link For code: Link to TRACE source code: https://gitlab.lrz.de/trace. Installation guide: https://gitlab.lrz.de/trace/trace-docs/-/wikis/TACE/Installation.

## Abbreviations

AI: Artificial Intelligence
API: Application Programming Interface
CONSORT: Consolidated Standards of Reporting Trials
CPU: Central Processing Unit
CSV: Comma-Separated Values
DOI: Digital Object Identifier
FAIR: Findable, Accessible, Interoperable, Reusable
FHIR: Fast Healthcare Interoperability Resources
GDPR: General Data Protection Regulation
ICD: International Classification of Diseases
IT: Information Technology
JSON: JavaScript Object Notation
JSON-LD: JavaScript Object Notation for Linked Data
KVM: Kernel-based Virtual Machine
LDAP: Lightweight Directory Access Protocol
LOINC: Logical Observation Identifiers Names and Codes
LRZ: Leibniz-Rechenzentrum
QWD: QEMU-Web-Desktop
QEMU: Quick Emulator
REDCap: Research Electronic Data Capture
SNOMED CT: Systematized Nomenclature of Medicine Clinical Terms
SOP: Standard Operating Procedure
SPIRIT: Standard Protocol Items: Recommendations for Interventional Trials
SQL: Structured Query Language
SSH: Secure Shell
TLS: Transport Layer Security
TRACE: Trusted Research Access & Collaboration Environment
TRE: Trusted Research Environment
TRUST: Transparency, Responsibility, User focus, Sustainability, and Technology
VNC: Virtual Network Computing
VM: Virtual Machine
XML: Extensible Markup Language
YODA: Yale Open Data Access
