# Supplementary material for: FAIR by design (TRACE): A Trusted Research Access & Collaboration Environment
Source: BMC Med Inform Decis Mak. 2026 Sep 15;26:350. doi: 10.1186/s12911-026-03840-3 (PMC13576130; doi:10.1186/s12911-026-03840-3)
Supplement: Supplementary file 1 [file 12911_2026_3840_MOESM1_ESM.docx]

# Electronic supplementary material

**Additional File 1:** Extensible Connector Architecture. JSON metadata format which can be attached to any project in the TRACE. Recommended are file-based projects, for instance if the study data is hold as a CSV file**.**

{

"file_path": "/path/to/school_health.csv",

"delimiter": ",",

"events": {

"StartOfYear": {

"instruments": {

"anthro": [

"anthro_height",

"anthro_weight"

],

"vision": [

"vision_left_eye",

"vision_right_eye"

]

}

},

"EndOfYear": {

"instruments": {

"anthro": [

"anthro_height",

"anthro_weight"

],

"vision": [

"vision_left_eye",

"vision_right_eye"

]

}

}

},

"variables": {

"anthro_height": "Height (cm)",

"anthro_weight": "Weight (kg)",

"vision_left_eye": "Left Eye Acuity",

"vision_right_eye": "Right Eye Acuity"

}

}

**Additional File 2:** FAIR Maturity Indicator self-assessment, assessed on 27 Jul 2026. The table records the state of the platform at that date and is re-scored whenever capabilities change. ✓ implemented; ~ partly implemented, or dependent on the data-owner configuration; ✗ not implemented at the current stage of development.

| **FAIR ID** | **Indicator** | **Priority** | **Compliance** | **Notes** |
| --- | --- | --- | --- | --- |
| **F1-01M** | Metadata is identified by a persistent identifier | Essential | ✓ | Every study and variable in TRACE has a stable, unique URI. |
| **F1-01D** | Data is identified by a persistent identifier | Essential | ~ | Approved dataset extracts receive stable internal application identifiers; globally registered identifiers are planned. |
| **F1-02M** | Metadata is identified by a globally unique identifier | Essential | x | DOI can be added to the metadata in approx. Q1 2027 |
| **F1-02D** | Data is identified by a globally unique identifier | Essential | ~ | Internal unique IDs exist; plan to register DOIs for published extracts. |
| **F2-01M** | Rich metadata is provided to allow discovery | Essential | ✓ | Full variable‐level descriptions, labels, data dictionaries, custom codebook metadata. |
| **F3-01M** | Metadata includes the identifier for the data | Essential | ✓ | Each metadata record references the corresponding dataset by ID or study title. |
| **F4-01M** | Metadata is offered for harvesting and indexing | Essential | ✓ | Metadata can be freely harvested |
| **A1-01M** | Metadata contains information to enable the user to get access to data | Important | ✓ | For each study a direct data access application form is provided |
| **A1-02M** | Metadata can be accessed manually (human intervention) | Essential | ✓ | All metadata browsable via web UI. |
| **A1-02D** | Data can be accessed manually (human intervention) | Essential | ✓ | Self‐service access of approved extracts within TRE. |
| **A1-03M** | Metadata identifier resolves to a metadata record | Essential | ✓ | URIs resolve to JSON or HTML metadata views. |
| **A1-03D** | Data identifier resolves to a digital object | Essential | ~ | Stable application IDs are linked to archived CSV and metadata files; a dedicated protected resolver for the dataset object is not yet implemented. |
| **A1-04M** | Metadata is accessed through standardized protocol | Essential | ✓ | Through HTTPS; plan to support SPARQL/OAI‐PMH for broader uptake. |
| **A1-04D** | Data is accessible through standardized protocol | Essential | ✓ | HTTPS access endpoints embedded within TRE. |
| **A1-05D** | Data can be accessed automatically (computer program) | Important | ✓ | Fully supported programmatic access within the secured institutional TRE via RStudio, Jupyter Notebook, or other authorised intranet tools. |
| **A1.1-01M** | Metadata accessible via free access protocol | Essential | ✓ | Public metadata browsable with no login. |
| **A1.1-01D** | Data accessible via free access protocol | Important | ✓ | Data access requires authenticated approval; this is intended. |
| **A1.2-01D** | Data accessible via protocol supporting authentication/authorization | Useful | ✓ | LDAP‐backed login for TRE environment. |
| **A2-01M** | Metadata guaranteed to remain available after data is gone | Essential | ~ | Metadata retention policy exists; archival module planned with the university library. |
| **I1-01M** | Metadata uses knowledge representation in standardized format | Important | ✓ | Via HTML and JSON |
| **I1-02M** | Metadata uses machine‐understandable knowledge representation | Important | ✓ | Metadata of a study can be publicly available in JSON-LD, which is accomplished automatically by publishing a REDCap study. |
| **I2-01M** | Metadata uses FAIR‐compliant vocabularies | Important | ~ / ✓ | Depends on the data owner who developed the study |
| **I3-01M** | Metadata includes references to other metadata | Important | ✓ | REDCap: data dictionary  File based: config file Other sources: APIs |
| **R1-01M** | Plurality of accurate, relevant attributes for reuse | Essential | ✓ | Detailed variable labels, descriptions |
| **R1.1-01M** | Metadata includes license info for reuse | Essential | ~ | Planned |
| **R1.3-01M** | Metadata complies with community standard | Essential | ~ | Depends on data owner |
| **R1.3-02M** | Metadata machine‐understandable community standard | Essential | ✓ | JSON‐LD export of metadata is implemented. |

**Additional File 3:** Example JSON-LD metadata snippet (PEACHES dataset). This excerpt illustrates the machine-readable metadata structure (schema.org/Dataset). The full JSON-LD file for PEACHES is available at:

https://trace.ibe.med.uni-muenchen.de/harvest/project/1/metadata.json

{

"@context": "https://schema.org",

"@type": "Dataset",

"name": "PEACHES",

"description": "Metadata record for the clinical research dataset of the study “PEACHES”, curated in the TRACE research data platform. It describes the data structure of a longitudinal study with 11 events, comprising 2366 variables.",

"identifier": [

{

"@type": "PropertyValue",

"propertyID": "REDCap project ID",

"value": "24"

}

],

"url": "https://trace.ibe.med.uni-muenchen.de/harvest/project/1/metadata.json",

"dateCreated": "2025-02-07T17:25:14.383459+00:00",

"dateModified": "2026-07-22T13:05:19.847182+00:00",

"keywords": [

"foetal programming",

"gestational diabetes",

"obesity",

"cardiometabolic risk",

"maternal metabolic environment",

"child development",

"longitudinal study"

]

}

**Additional File 4**: Derivation of export-event metrics

This file documents how the export figures reported in the Results section were derived from REDCap event logs so that the analysis can be checked and reproduced. It specifies the definitions, observation windows, data sources, inclusion and exclusion criteria, per-project results, limitations, provenance, and queries used.

**Definitions**

**Manual interface export**. A log row with event = 'DATA_EXPORT' and page = 'DataExport/data_export_ajax.php', operationally defined as an export requested through the REDCap web interface. This is the figure reported in the manuscript. The page value is matched exactly; events with any other page value are classified as other and are not counted as manual interface exports.

**API-associated export event**. A log row with event = 'DATA_EXPORT' and a page matching api/%. These events are classified separately, are never merged into the manual figure, and are not interpreted as a count of provisioned datasets (see section 6).

**Counting unit**. Counts represent export events, not distinct datasets or distinct users. A record set exported repeatedly is counted once on each occasion. An export event records that REDCap processed an export action on that occasion. It does not establish that the resulting file was successfully downloaded, how much data it contained, whether it was subsequently used, or the sensitivity of the fields involved.

**Observation windows**

TRACE went live on 18 March 2025. Two primary observation windows and one extended sensitivity window are defined:

| **Window** | **Start, inclusive** | **End, exclusive** | **Days** |
| --- | --- | --- | --- |
| Pre-implementation | 1 Jan 2022 | 18 Mar 2025 | 1,172 |
| Post-implementation | 18 Mar 2025 | 1 Jan 2026 | 289 |
| Post, extended sensitivity analysis | 18 Mar 2025 | 1 Jul 2026 | 470 |

The bounds are half-open. An event belongs to the pre-implementation period if its timestamp is at or after 1 January 2022 and before 18 March 2025. It belongs to the post-implementation period if its timestamp is at or after 18 March 2025 and before 1 January 2026. The go-live boundary is applied at midnight on 18 March 2025 because the exact deployment time within that day was not recorded. Rates are expressed per 30 days and are calculated as the number of events divided by the number of days in the window, multiplied by 30. This permits comparison between windows of different lengths; the pre-implementation period is approximately four times as long as the primary post-implementation period.

**Data sources**

The three studies onboarded to TRACE span four REDCap projects on two separate REDCap instances. Each project writes to its own log shard. Project identifiers are unique only within a host; consequently, the two instances must be queried separately and cannot be joined directly within a single REDCap Query Tool query.

| **Study or project** | **Host** | **Project ID** | **Log table** |
| --- | --- | --- | --- |
| PEACHES | redcap | 24 | redcap_log_event7 |
| BEARR | edc | 125 | redcap_log_event2 |
| BEARR Randomization, excluded | edc | 126 | redcap_log_event3 |
| ENABLE | edc | 129 | redcap_log_event5 |

The generic table redcap_log_event returned no rows for these projects during this analysis. The project-specific log shard must therefore be resolved first using queries Q1a and Q1b.

**Inclusion and exclusion criteria**

Three sets of events were excluded, each for a stated reason.

**Randomization project, project 126**. Exports from the randomization project contain allocation data rather than research datasets and are governed by a different process. Including this project would add 97 events to the pre-implementation period and one event to the post-implementation period.

**Events before 1 January 2022**. A common starting date was applied to all three studies. The ENABLE log begins on 6 May 2021 and contains 88 earlier manual interface exports. These events fall outside the common observation window and are not counted.

**API Playground events during TRACE build-out**. Fifty-two events on PEACHES and two on ENABLE, recorded during February and March 2025, represent developer testing during implementation. These are API-associated events and therefore fall outside the manual interface export definition in any case.

**Results**

Manual interface exports per project were as follows:

| **Study** | **Pre, 1,172 days** | **Post, 289 days** |
| --- | --- | --- |
| PEACHES | 537 | 27 |
| BEARR | 613 | 9 |
| ENABLE | 505 | 9 |
| **Total** | **1,655** | **45** |

Pooled across the three studies, this corresponds to 42.4 manual interface exports per 30 days before implementation—1,655 events over 1,172 days—and 4.7 per 30 days after implementation—45 events over 289 days. This represents a decrease of 89.0%.

Sensitivity to the endpoint. Extending the post-implementation period to 1 July 2026 gives 47 manual interface exports over 470 days, corresponding to 3.0 per 30 days. This represents a decrease of 92.9% relative to the pre-implementation rate.

Sensitivity to the randomization-project exclusion. Including project 126 would give 1,752 pre-implementation events and 46 post-implementation events, corresponding to 44.8 and 4.8 events per 30 days, respectively. This represents a decrease of 89.3%. The exclusion is based on the different function and governance of the randomization project and does not materially affect the result.

**Why API events are not reported as a provisioning count**

API-associated events recorded under the TRACE service accounts arise from two distinct operations that cannot be separated using the page field alone:

1. retrieval of study metadata to render the catalogue and study visualisation whenever a study page is viewed; and
2. extraction of an approved dataset when an application is provisioned.

Reporting the combined number as a count of provisioning operations would therefore overstate provisioning.

A further 87 API-associated events on PEACHES after go-live were recorded under the administrator account and could not be attributed to either operation.

For these reasons, no API-derived provisioning count is reported in the manuscript. The reported figures include only manual interface exports and are unaffected by this ambiguity.

**Note on the previously reported figure**

An earlier version of the manuscript reported 1,707 manual exports during the pre-implementation period. Because the query underlying that figure was not retained, the result could not be reproduced or fully audited.

Re-analysis using the explicit definitions and queries reported here yielded 1,655 manual interface export events for the same observation window. The earlier figure is therefore superseded by the reproducible result.

Comparison with the current event inventory suggests that API-associated events may account for some or all of the discrepancy. Because the original query and its outputs were not retained, the source of the difference cannot be established conclusively.

**Limitations of this derivation**

Rates are pooled across the three studies over calendar time and are not adjusted for staggered project activity. Where a project was not active throughout the entire pre-implementation period, the pooled pre-implementation rate is correspondingly conservative.

Export volume may vary with the lifecycle stage of a study independently of any platform change. The comparison is therefore an uncontrolled before-and-after observation and does not support causal attribution.

The logs record export actions performed within the source REDCap systems only. Exports performed outside REDCap and onward transfers of data that had already been extracted are not observable in these data.

**Provenance**

The queries were initially executed on 23 July 2026 through the REDCap Query Tool using read-only access to the production databases of the two REDCap instances, hosts redcap and edc, running MariaDB.

All host-specific queries listed in section 10 were rerun for verification on 28 July 2026, and their outputs were retained as dated CSV files. The verification outputs reproduced the project-to-log-table mappings, event inventories, account and page classifications, pre- and post-implementation counts, and monthly series reported in this file. The extended-period total was also confirmed from the post-go-live exporter queries: 29 manual interface exports on host redcap and 18 on host edc, giving 47 events in total.

No record-level study data and no export files were retrieved. The analysis used aggregated audit-log metadata only: event counts, page values, log-event descriptions, account names, and date ranges. Account names were used for attribution during the analysis and are not reported in the manuscript or in this file.

**Queries**

REDCap's Query Tool accepts only statements beginning with SELECT, SHOW, or EXPLAIN. Common table expressions beginning with WITH are rejected, so unions are placed inside derived tables. Trailing semicolons and inline comments are omitted for the same reason.

The alias ym is used in the monthly-series queries because YEAR_MONTH is a reserved MariaDB keyword.

**Q1a: Resolve the log table on host redcap**

SELECT project_id, app_title, log_event_table

FROM redcap_projects

WHERE project_id = 24

**Q1b: Resolve the log tables on host edc**

SELECT project_id, app_title, log_event_table

FROM redcap_projects

WHERE project_id IN (125, 126, 129)

**Q2: Event inventory on host redcap—PEACHES**

SELECT page,

description,

COUNT(*) AS n,

MIN(ts) AS first_ts,

MAX(ts) AS last_ts

FROM redcap_log_event7

WHERE project_id = 24

AND event = 'DATA_EXPORT'

GROUP BY page, description

ORDER BY n DESC

**Q3: Event inventory on host edc—BEARR, BEARR Randomization, and ENABLE**

SELECT project_id,

page,

description,

COUNT(*) AS n,

MIN(ts) AS first_ts,

MAX(ts) AS last_ts

FROM (

SELECT project_id, page, description, ts

FROM redcap_log_event2

WHERE project_id = 125

AND event = 'DATA_EXPORT'

UNION ALL

SELECT project_id, page, description, ts

FROM redcap_log_event3

WHERE project_id = 126

AND event = 'DATA_EXPORT'

UNION ALL

SELECT project_id, page, description, ts

FROM redcap_log_event5

WHERE project_id = 129

AND event = 'DATA_EXPORT'

) AS ev

GROUP BY project_id, page, description

ORDER BY project_id, n DESC

**Q4: Post-go-live exporters on host redcap, 18 March 2025 to 1 July 2026**

SELECT `user`,

page,

COUNT(*) AS n,

MIN(ts) AS first_ts,

MAX(ts) AS last_ts

FROM redcap_log_event7

WHERE project_id = 24

AND event = 'DATA_EXPORT'

AND ts >= 20250318000000

AND ts < 20260701000000

GROUP BY `user`, page

ORDER BY n DESC

**Q5: Post-go-live exporters on host edc, 18 March 2025 to 1 July 2026**

SELECT project_id,

`user`,

page,

COUNT(*) AS n,

MIN(ts) AS first_ts,

MAX(ts) AS last_ts

FROM (

SELECT project_id, `user`, page, ts

FROM redcap_log_event2

WHERE project_id = 125

AND event = 'DATA_EXPORT'

AND ts >= 20250318000000

AND ts < 20260701000000

UNION ALL

SELECT project_id, `user`, page, ts

FROM redcap_log_event3

WHERE project_id = 126

AND event = 'DATA_EXPORT'

AND ts >= 20250318000000

AND ts < 20260701000000

UNION ALL

SELECT project_id, `user`, page, ts

FROM redcap_log_event5

WHERE project_id = 129

AND event = 'DATA_EXPORT'

AND ts >= 20250318000000

AND ts < 20260701000000

) AS ev

GROUP BY project_id, `user`, page

ORDER BY project_id, n DESC

**Q6: Pre- versus post-implementation counts on host redcap—PEACHES**

SELECT CASE

WHEN ts < 20250318000000 THEN 'pre'

ELSE 'post'

END AS period,

CASE

WHEN page = 'DataExport/data_export_ajax.php' THEN 'manual_ui'

WHEN page LIKE 'api/%' THEN 'api'

ELSE 'other'

END AS export_type,

COUNT(*) AS n_exports

FROM redcap_log_event7

WHERE project_id = 24

AND event = 'DATA_EXPORT'

AND ts >= 20220101000000

AND ts < 20260101000000

GROUP BY period, export_type

ORDER BY period, export_type

**Q7: Pre- versus post-implementation counts on host edc**

This query includes the excluded randomization project, project 126, so that the exclusion can be checked.

SELECT project_id,

CASE

WHEN ts < 20250318000000 THEN 'pre'

ELSE 'post'

END AS period,

CASE

WHEN page = 'DataExport/data_export_ajax.php' THEN 'manual_ui'

WHEN page LIKE 'api/%' THEN 'api'

ELSE 'other'

END AS export_type,

COUNT(*) AS n_exports

FROM (

SELECT project_id, ts, page

FROM redcap_log_event2

WHERE project_id = 125

AND event = 'DATA_EXPORT'

UNION ALL

SELECT project_id, ts, page

FROM redcap_log_event3

WHERE project_id = 126

AND event = 'DATA_EXPORT'

UNION ALL

SELECT project_id, ts, page

FROM redcap_log_event5

WHERE project_id = 129

AND event = 'DATA_EXPORT'

) AS ev

WHERE ts >= 20220101000000

AND ts < 20260101000000

GROUP BY project_id, period, export_type

ORDER BY project_id, period, export_type

Q6 and Q7 as written cover 1 January 2022 to 1 January 2026. For the extended post-implementation period reported in section 5, change the upper bound in both queries to:

20260701000000

Both hosts must use the same upper bound. The reported manual figures are the manual_ui rows. Any returned other rows must be inspected and must not be treated as manual interface exports.

**Q8a: Monthly series of manual interface exports on host redcap**

SELECT project_id,

LEFT(CAST(ts AS CHAR), 6) AS ym,

COUNT(*) AS n_exports

FROM redcap_log_event7

WHERE project_id = 24

AND event = 'DATA_EXPORT'

AND page = 'DataExport/data_export_ajax.php'

AND ts >= 20220101000000

AND ts < 20260701000000

GROUP BY project_id, ym

ORDER BY project_id, ym

**Q8b: Monthly series of manual interface exports on host edc**

SELECT project_id,

LEFT(CAST(ts AS CHAR), 6) AS ym,

COUNT(*) AS n_exports

FROM (

SELECT project_id, ts, page

FROM redcap_log_event2

WHERE project_id = 125

AND event = 'DATA_EXPORT'

UNION ALL

SELECT project_id, ts, page

FROM redcap_log_event5

WHERE project_id = 129

AND event = 'DATA_EXPORT'

) AS ev

WHERE page = 'DataExport/data_export_ajax.php'

AND ts >= 20220101000000

AND ts < 20260701000000

GROUP BY project_id, ym

ORDER BY project_id, ym
